# d-Galactose Decreases Anion Exchange Capability through Band 3 Protein in Human Erythrocytes

**DOI:** 10.3390/antiox9080689

**Published:** 2020-08-02

**Authors:** Alessia Remigante, Rossana Morabito, Sara Spinelli, Vincenzo Trichilo, Saverio Loddo, Antonio Sarikas, Silvia Dossena, Angela Marino

**Affiliations:** 1 Department of Chemical, Biological, Pharmaceutical and Environmental Sciences, University of Messina, Viale F. Stagno D’Alcontres 31, 98166 Messina, Italy; aremigante@unime.it (A.R.); rmorabito@unime.it (R.M.); sara.spinelli1992@libero.it (S.S.); 2 Department of Clinical and Experimental Medicine, AOU Policlinico Universitario “G. Martino”, Via Consolare Valeria, 98125 Messina, Italy; vtrichilo@unime.it (V.T.); sloddo@unime.it (S.L.); 3 Institute of Pharmacology and Toxicology, Paracelsus Medizinische Privatuniversität, Strubergasse 21, Haus C, 5020 Salzburg, Austria; antonio.sarikas@pmu.ac.at (A.S.); silvia.dossena@pmu.ac.at (S.D.)

**Keywords:** d-Galactose, oxidative stress, glycation, Band 3 protein, SO_4_^2−^ uptake, anion exchange

## Abstract

d-Galactose (d-Gal), when abnormally accumulated in the plasma, results in oxidative stress production, and may alter the homeostasis of erythrocytes, which are particularly exposed to oxidants driven by the blood stream. In the present investigation, the effect of d-Gal (0.1 and 10 mM, for 3 and 24 h incubation), known to induce oxidative stress, has been assayed on human erythrocytes by determining the rate constant of SO_4_^2−^ uptake through the anion exchanger Band 3 protein (B3p), essential to erythrocytes homeostasis. Moreover, lipid peroxidation, membrane sulfhydryl groups oxidation, glycated hemoglobin (% A1c), methemoglobin levels (% MetHb), and expression levels of B3p have been verified. Our results show that d-Gal reduces anion exchange capability of B3p, involving neither lipid peroxidation, nor oxidation of sulfhydryl membrane groups, nor MetHb formation, nor altered expression levels of B3p. d-Gal-induced %A1c, known to crosslink with B3p, could be responsible for rate of anion exchange alteration. The present findings confirm that erythrocytes are a suitable model to study the impact of high sugar concentrations on cell homeostasis; show the first in vitro effect of d-Gal on B3p, contributing to the understanding of mechanisms underlying an in vitro model of aging; demonstrate that the first impact of d-Gal on B3p is mediated by early Hb glycation, rather than by oxidative stress, which may be involved on a later stage, possibly adding more knowledge about the consequences of d-Gal accumulation.

## 1. Introduction

d-Galactose (d-Gal) is a monosaccharide sugar, whose metabolism is connected to glucose metabolism [1]. In physiological conditions, it is rapidly metabolized to glucose through specific enzymes and driven from peripheral blood to glycolysis pathway to give energy to the cell [2], keeping thus plasma d-Gal levels very low (plasmatic concentration range between 0.00008 and 0.00018 mM) [3,4]. Abnormally accumulated d-Gal [5,6], associated to galactosemia, can be directly oxidized by galactose oxidase to produce hydrogen peroxide (H_2_O_2_) or, alternatively, can start a non-enzymatic glycation reactions with free amino acid (mainly lysine and arginine) to form advanced glycation end products (AGEs) [7,8,9].

When oxidative stress persists, the excessive free radical species accumulation can influence many cellular signaling pathways and damage organic macromolecules, which can be oxidized [10,11]. The overproduction of oxidative stress is a common patho-physiological state underlying many chronic diseases, such as cardiovascular diseases [12,13], neurodegenerative diseases [14], cancer [15], metabolic disorders (diabetes mellitus [16]), and diseases related to aging [17], although the precise mechanisms contributing to the oxidative stress-induced damage are still under investigation. Several studies have exploited the in vivo exposure to d-Gal as a typical model for exploring the mechanisms underlying oxidative stress-related diseases in plasma or blood cells [6,18,19,20,21]. However, the effects of d-Gal on oxidative stress production in human erythrocytes have been only partially clarified [22]. Moreover, it has been established that high sugar concentrations are correlated with an increase in glycation of human erythrocytes proteins, resulting in modifications of lipids-proteins interaction. Glycation of membrane proteins could induce membrane fluidity alteration, which reflects not only on membrane bilayer, but also on intracellular environment [22,23,24,25].

Human erythrocytes are continuously exposed to oxidative molecules transported within the vascular system and possibly interacting with erythrocytes membrane, and are more susceptible to oxidative stress than other cells [26]. Oxidative damage has been demonstrated to decrease survival and rheological properties of human erythrocytes, affecting their homeostasis, which is strictly linked to Band 3 protein (B3p) function [27,28]. B3p mediates the Cl^−^/HCO_3_^−^ exchange across erythrocytes membrane and plays a key role for erythrocytes deformability, ion balance and gas exchange, all essential to tissue oxygenation [29,30]. The uptake of SO_4_^2−^, slower and better detectable than that of Cl^−^ or HCO_3_^−^ [31,32], has been used to monitor the anion exchange capability through this protein by a turbidimetric method. In particular, this method has been effectively used in vitro to investigate the impact of different conditions on human erythrocytes homeostasis, including H_2_O_2_-induced oxidative conditions, oxidative stress-related diseases, or high glucose levels [33,34,35], proving that anion exchange capability of B3p is a suitable tool to evaluate cell response to oxidative conditions. In addition, Pantaleo and collaborators [36] demonstrated that erythrocytes possess a mechanism to sense the oxidative stress via B3p oxidation, docking of Syk kinase and phosphorylation of two B3p Tyr residues, crucial for assuring membrane stability.

The effect of d-Gal on B3p function has never been evaluated. Since an excessive exposure to d-Gal (25 mM) could lead to the overproduction of reactive oxygen species (ROS) and AGEs in erythrocytes, thus contributing to oxidative stress events [22], and considering the current scarce availability of studies in this field, in the present investigation we firstly tried to detect a possible early damage of erythrocyte homeostasis by measuring the anion exchange capability through B3p following short-term exposure (3 and 24 h) of human erythrocytes to 0.1 and 10 mM d-Gal [6] and, secondarily, to verify whether at that stage the potential alterations may be linked to redox balance variations. The present study is included in a wider field of investigation about the relationship, still poorly clarified, between high d-Gal concentrations and membrane transport systems.

## 2. Materials and Methods

### 2.1. Solutions and Chemicals

The specific catalase inhibitor 3-amino-1,2,4-triazole (3-AT) [37], freshly prepared H_2_O_2_ and NaNO_2_ were dissolved in distilled water and diluted from 3 M, 50 mM and 30% *w/w* stock solutions, respectively. 4,4′-diisothiocyanato-stilbene-2,2′-disulfonate (DIDS) was prepared in dimethyl sulfoxide (DMSO) and diluted from a 10 mM stock solution. N-ethylmaleimide (NEM) was prepared in ethanol and diluted from a 310 mM stock solution. All chemicals were purchased from Sigma (Milan, Italy). DMSO and ethanol were tested on erythrocytes at their final concentration (0.001% *v/v*) to preventively exclude any effect related to the solvent.

### 2.2. Erythrocytes Preparation

**Figure 1:**
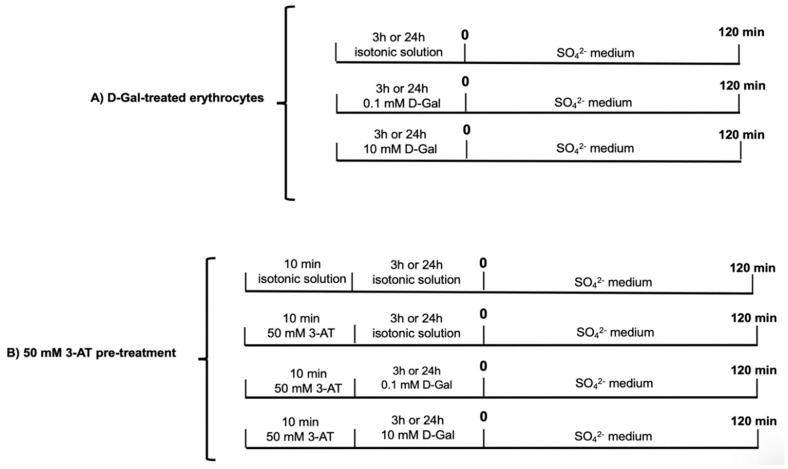
Time course of different experimental conditions before SO_4_^2−^ uptake measurement. (**A**) Gal-treated erythrocytes (0.1 or 10 mM, for 3 or 24 h). (**B**) 50 mM 3-AT pretreatment (10 min) plus Gal-treatment.

Blood samples were obtained after oral informed consent from adults healthy volunteers (13 ♀, 11 ♂, age 28–45 years) with blood d-Gal concentrations ranging between 0.00008 and 0.00018 mmol/L (physiological concentrations) [4]. Patients with diabetes and cardiovascular disease have been excluded from the study. Blood, collected in tubes containing ethylenediaminetetraacetic acid (EDTA) to prevent coagulation, was washed with an isotonic solution with the following composition (in mM): NaCl 145, glucose 5,4-(2-hydroxyethyl)-1 piperazineethanesulfonic acid (HEPES) 5, pH 7.4, osmotic pressure 300 mOsm/KgH_2_O). The samples were centrifuged thrice (ThermoScientific, Milan, Italy, 1200× *g*, 5 min) to discard both plasma and buffy coat. Subsequently, erythrocytes were suspended in isotonic solution at different hematocrit values and addressed to different treatments according to the experimental design reported in Figure 1 (see treatments before time 0). After each treatment, erythrocytes were subjected to SO_4_^2−^ uptake measurement, oxidative condition assessment, Western blot analysis, or glycated hemoglobin measurement.

### 2.3. SO_4_^2−^ Uptake Measurement

#### 2.3.1. Control Condition

SO_4_^2−^ uptake via B3p was measured as described elsewhere [31,38]. After washing, erythrocytes samples were suspended to 3% hematocrit and addressed to either control conditions assessment or to incubation with d-Gal. With regard to control conditions, erythrocytes were suspended in 35 mL isotonic solution containing SO_4_^2−^, henceforth called SO_4_^2−^ medium (composition in mM: Na_2_SO_4_ 118, HEPES 10, glucose 5, pH 7.4, osmotic pressure 300 mOsm/KgH_2_O) and incubated at 25 °C. At fixed time intervals (5, 10, 15, 30, 45, 60, 90 and 120 min), 5 mL erythrocytes suspension were transferred in a tube containing DIDS (10 μM) to block B3p activity [39], and kept on ice. After incubation in SO_4_^2−^ medium plus DIDS, samples were washed at least thrice by centrifugation (ThermoScientific, 4 °C, 1200× *g*, 5 min) and resuspension in isotonic solution to eliminate SO_4_^2−^ from the external medium, and then hemolyzed in distilled water (1 mL). Proteins were precipitated by perchloric acid (4% *v/v*). After centrifugation (ThermoScientific 4 °C, 2500× *g*, 10 min), the supernatant containing SO_4_^2−^ underwent turbidimetric analysis. SO_4_^2−^ precipitation was performed by mixing the following components: 500 μL supernatant from each sample, 1 mL glycerol previously diluted in distilled water (1:1), 1 mL 4 M NaCl plus 37% hydrochloric acid (HCl) solution (12:1) and, finally, 500 μL 1.24 M BaCl_2_·2H_2_O. The absorbance of each sample was then read at 425 nm using a spectrophotometer (Eppendorf, BioPhotometer Plus, Milan, Italy).

A calibrated standard curve previously obtained by precipitating known SO_4_^2−^ concentrations was used to convert the absorbance to [SO_4_^2−^] L cells × 10^−2^. Moreover, the rate constant (min^−1^) was calculated by the equation: C_t_ = C_∞_ (1 − e^−*rt*^) + C_0_, where C_t_, C_∞_ and C_0_ indicate the intracellular SO_4_^2−^ concentrations at time t, 0 and ∞ respectively, e is Neper number (2.7182818), *r* is the rate constant accounting for the process velocity and *t* is the time of each sample withdrawal. The rate constant is the time needed to reach 63% of total SO_4_^2−^ intracellular concentration [31] and [SO_4_^2−^] L cells × 10^−2^ reported in figures stands for SO_4_^2−^ micromolar concentration trapped by 5 mL erythrocytes (3% hematocrit).

#### 2.3.2. d-Gal-Treated Erythrocytes

Once washed and suspended to 3% hematocrit and after either 3 or 24 h incubation at different d-Gal concentrations added to the solution (0.1 and 10 mM, at 25 °C), erythrocytes samples were washed and centrifuged (ThermoScientific, 4 °C, 1200× *g*, 5 min) to replace the supernatant with SO_4_^2−^ medium. SO_4_^2−^ uptake was successively determined as described for control conditions. In addition, to assay whether d-Gal effect was due to antioxidant system failure, 3-amino-1,2,4-triazole (3-AT), a specific inhibitor of catalase [37], was applied 10 min before both 3 and 24 h treatment with either 0.1 or 10 mM d-Gal. In Figure 1, the time course of all experimental conditions followed by SO_4_^2−^ uptake determination is shown.

### 2.4. Oxidative Condition Assessment

#### 2.4.1. Thiobarbituric Acid Reactive Species Determination

To assess a possible lipid peroxidative effect of d-Gal on erythrocytes membranes, thiobarbituric acid reactive species (TBARS) levels, deriving from reaction between thiobarbituric acid (TBA) and malondialdehyde (MDA), i.e., the end products of the lipid peroxidation, were measured as described by Mendanha and collaborators [40], with slight modifications. Trichloroacetic acid (TCA, final concentration 10% *w/v*) was added to 1.5 mL of erythrocytes, which were either untreated or pre-treated with d-*G*al with or without 3-AT (Figure 1) and suspended at 20% hematocrit. Samples underwent centrifugation (ThermoScientific, 25° C, 3000× *g*, 10 min) and 1 mL TBA (1% in 0.05 M NaOH) was added to the supernatant. Then, the mixture was heated for 30 min to 95 °C. Finally, TBARS levels were obtained by subtracting 20% of the absorbance at 453 nm from the absorbance at 532 nm (Eppendorf, BioPhotometer Plus). Erythrocytes samples were also incubated with H_2_O_2_ (10 mM, for 1 h, at 25 °C), as this compound is known to induce a significant lipid peroxidative effect [41]. Results are indicated as μM TBARS levels (1.56 × 10^5^ M^−1^ cm^−1^ molar extinction coefficient).

#### 2.4.2. Membrane Sulfhydryl Groups Levels Determination

Membrane sulfhydryl groups concentration was estimated by the method of Aksenov and Markesbery [42], with some modifications. Shortly, 100 μL of washed erythrocytes, which were either untreated, or pre-treated with d-Gal with or without 3-AT (Figure 1) and suspended at 35% hematocrit, were added to 1 mL of distilled water. Then, a 50 μL aliquot was diluted with 1 mL phosphate-buffered saline (PBS), pH 7.4, containing 1 mM EDTA. The addition of 30 μL of 10 mM 5,5′-dithiobis-(2-nitrobenzoic acid) (DTNB) began the reaction and samples were incubated for 30 min in a dark room at 25 °C. Control samples, without protein extract or DTNB, were simultaneously handled. Afterwards, samples were spectrophotometrically read at 412 nm (Eppendorf, BioPhotometer Plus), and levels of 3-thio-2-nitro-benzoic acid (TNB) determined by comparison to blank (DTNB absorbance). NEM (2 mM for 1 h incubation, at 25 °C) was used to obtain complete oxidation of membrane sulfhydryl groups [27,43]. Results are reported as μM TNB/mg protein and data were normalized to protein content.

#### 2.4.3. Methemoglobin Levels Determination

Methemoglobin (MetHb) levels have been assayed as previously described [44,45] with some modifications. This assay is based on methemoglobin and (oxy)-hemoglobin determination by spectrophotometry at, respectively, 630 and 540 nm wavelengths. Shortly, 25 μL of erythrocytes at 35% hematocrit, which were untreated or pre-treated d-Gal with or without 3-AT (Figure 1), were lysed in 1975 μL hypotonic buffer (2.5 mM NaH_2_PO_4_, pH 7.4, 4 °C). Erythrocytes samples were then centrifuged (Eppendorf, 4 °C, 13,000× *g*, 15 min) to discard membranes and the absorbance of the supernatant was read by a spectrophotometer (Eppendorf, BioPhotometer Plus). NaNO_2_ (4 mM for 1 h incubation at 25 °C) was used to obtain complete Hb oxidation [46]. MetHb percentage (%) was calculated as follows: %MetHb = OD_630_/OD_540_ × 100 (OD is optical density).

### 2.5. Erythrocytes Membranes Preparation

Erythrocyte membranes were prepared as described by other authors [36], with slight modifications. Briefly, packed erythrocytes (untreated or pre-treated with d-*G*al with or without 3-AT pre-treatment), were diluted into 1.5 mL of 2.5 mM NaH_2_PO_4_ (cold hemolysis solution) containing a cocktail of inhibitors (1 mM PMSF, 1 mM NaF, and 1 mM Na_3_VO_4_). Samples were repeatedly centrifuged (Eppendorf, 4 °C, 18,000× *g*, 10 min) to take out hemoglobin. The obtained membranes were solubilized by sodium dodecyl sulfate (SDS, 1% *v/v*) and kept for 20 min on ice. After centrifugation (Eppendorf, 4 °C, 13,000× *g*, 30 min), the supernatant, containing the solubilized membrane proteins, was stored at −80 °C until use.

### 2.6. SDS-PAGE Preparation and Western Blot Analysis

After thawing, membranes were solubilized in Laemmli buffer (1:1 volume ratio) [47] and heated for 10 min at 95 °C. The protein samples (20 μL) were separated by 7.5% SDS-polyacrylamide gel electrophoresis and transferred to a polyvinylidene fluoride membrane by applying a constant voltage (75 V) for 2 h at 4 °C. Membranes were blocked for 1 h at room temperature in 5% bovine serum albumin (BSA) diluted in Tris-buffered saline (150 mM NaCl, 15 mM Tris-HCl) containing 0.1% Tween-20 (TBST), and incubated overnight at 4 °C with the primary antibody (monoclonal anti-Band 3 protein antibody, B9277, Sigma-Aldrich, Milan, Italy, produced in mouse and diluted 1:5000 in TBST). Successively, membranes were incubated for 1 h with peroxidase-conjugated goat anti-mouse IgG secondary antibodies (A9044, Sigma-Aldrich, Milan, Italy) diluted 1:10,000 in TBST solution at room temperature. To assess the presence of equal amounts of protein, a monoclonal anti-actin antibody (A1978, Sigma-Aldrich, Milan, Italy), diluted 1:1000 in TBST solution and produced in mouse, was incubated with the same membrane, as suggested by Yeung and collaborators [48]. A chemiluminescence detection system (Super Signal West Pico Chemiluminescent Substrate, Pierce Thermo Scientific, Rockford, IL. USA) was used to detect signals, whose images were imported to analysis software (Image Quant TL, v2003). The intensity of the corresponding protein bands was determined by densitometry (Bio-Rad ChemiDocTM XRS+).

### 2.7. Glycated Hemoglobin Measurement (%A1c)

The levels of glycated hemoglobin (A1c) were determined with the A1c liquidirect reagent as previously described by Sompong and collaborators [24], with slight modifications. Erythrocytes samples (either untreated or pre-treated with d-Gal with or without 3-AT, Figure 1), were lysed with hemolysis buffer (2.5 mM NaH_2_PO_4_) and incubated with latex reagent at 37 °C for 5 min. The absorbance was measured at 610 nm (Eppendorf, BioPhotometer Plus). The levels of A1c were calculated from a standard curve obtained by using known concentrations of A1c, and expressed as %A1c.

### 2.8. Experimental Data and Statistics

Data are expressed as arithmetic means ± S.E.M. Statistical analysis was performed by the GraphPad Prism software (version 6.00 for Windows; San Diego, CA, USA). Significant differences between means were tested by one-way analysis of variance (ANOVA), followed by Bonferroni’s multiple comparison *post hoc* test. Statistically significant differences were assumed at *p* < 0.05; *n* represents the number of independent experiments.

## 3. Results

### 3.1. SO_4_^2−^ Uptake Measurement

**Figure 2:**
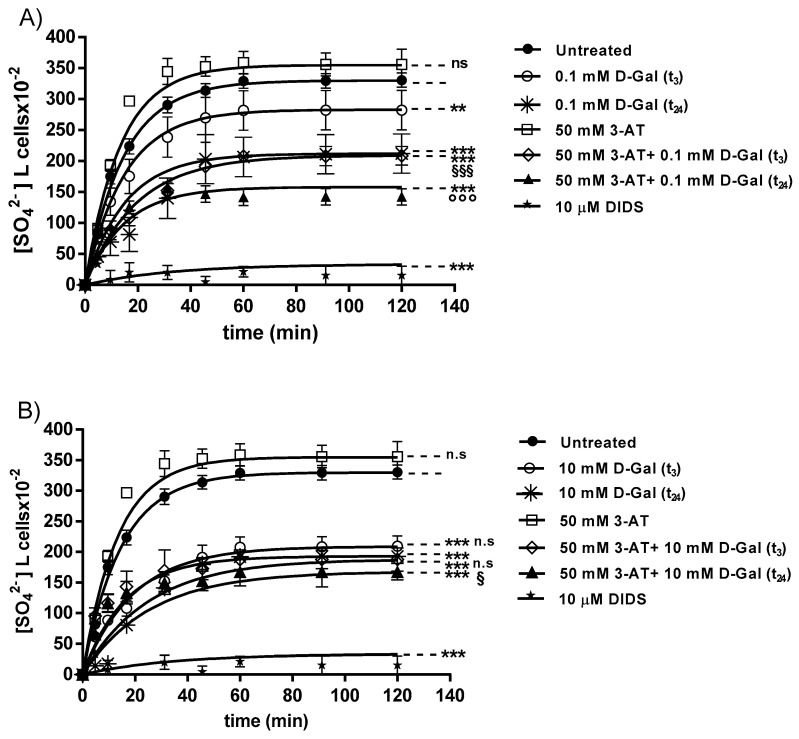
(**A**) Time course of SO_4_^2−^ uptake in untreated (control) and in 0.1 mM d-Gal (t_3_ and t_24_)-treated erythrocytes. ns, not significant versus control, ^***^ *p* < 0.001, ^**^, *p* < 0.01 versus control, ^§§§^, *p* < 0.001 versus 0.1 mM d-Gal (t_3_) and, ^°°°^, *p* < 0.001 versus 0.1 mM d-Gal (t_24_), as determined by one way ANOVA followed by Bonferroni’s post hoc test (n = 6). **(B**) Time course of SO_4_^2−^ uptake in untreated (control) and in 10 mM d-Gal (t_3_ and t_24_)-treated erythrocytes. ns, not significant versus control, ^***^, *p* < 0.001 versus control and ^§^, *p* < 0.05 versus 50 mM 3-AT + 10 mM d-Gal (t_3_), as determined by one way ANOVA followed by Bonferroni’s post hoc test (n = 6).

Figure 2A,B describe the SO_4_^2−^ uptake as a function of time in untreated erythrocytes (control) and erythrocytes treated with 0.1 or 10 mM d-Gal for 3 (t_3_) or 24 (t_24_) hours respectively, with or without pre-incubation with 50 mM 3-AT. In control conditions, the rate constant of SO_4_^2−^ uptake progressively increased and reached equilibrium within 45 min (0.067 ± 0.001 min^−1^). The rate constant values of SO_4_^2−^ uptake in d-Gal (0.1 or 10 mM)-treated erythrocytes at both t_3_ and t_24_ (0.066 ± 0.001 min^−1^, ^**^ *p* < 0.01; 0.065 ± 0.001 min^−1^, ^***^ *p* < 0.001 and 0.060 ± 0.001 min^−1^, ^***^ *p* < 0.001; 0.051 ± 0.001 min^−1^, ^***^ *p* < 0.001; Table 1) were significantly lower than control. The rate constant of SO_4_^2−^ uptake in erythrocytes treated with 50 mM 3-AT was not significantly different with respect to control (0.068 ± 0.001 min^−1^). After pretreatment of erythrocytes with 50 mM 3-AT and subsequent treatment with d-Gal, the rate constant of SO_4_^2−^ uptake was significantly lower than both control and d-Gal (0.1 or 10 mM) treatment (0.065 ± 0.001 min^−1^, ^***,§§§^, *p* < 0.001; 0.066 ± 0.001 min^−1^, ^***,°°°^, *p* < 0.001 and 0.040 ± 0.001 min^−1^, ns, ^***^, *p* < 0.001; 0.038 ± 0.001 min^−1^, ^***^, *p* < 0.001, ^§^, *p* < 0.05; Table 1). As expected, SO_4_^2−^ uptake was significantly blocked by 10 µM DIDS applied at the beginning of incubation in SO_4_^2−^ medium (0.018 ± 0.001 min^−1^, ^***^, *p* < 0.001, Table 1). The SO_4_^2−^ amount internalized by erythrocytes after 0.1 or 10 mM d-Gal treatment (t_3_ and t_24_) and 45 min of incubation in SO_4_^2−^ medium (269.76 ± 39.74 and 203.16 ± 39.74; 191.26 ± 19.90 and 179.97 ± 7.23, ^***^, *p* < 0.001, Table 1) was significantly lower with respect to that measured in control conditions (313.81 ± 11.09, Table 1). With regard to the treatment with 50 mM 3-AT alone, the amount of internalized SO_4_^2−^ was not different with respect to that measured in control conditions (343.43 ± 12.23), while with both 3-AT and 0.1 or 10 mM d-Gal pre-treatment, amounts of internalized SO_4_^2−^ were significantly lower than those measured following pre-treatment with 0.1 or 10 mM d-Gal alone (146.85 ± 18.46 and 191.26 ± 15.21, ^§§§,^***, *p* < 0.001; 172.85 ± 18 and 152.85 ± 15.87, ^§^, *p* < 0.05; Table 1). In DIDS-treated cells, the intracellular SO_4_^2−^ amount after 45 min of incubation in SO_4_^2−^ medium (4.75 ± 8.50) was significantly lower than that determined in control or in d-Gal (0.1 and 10 mM)-treated erythrocytes, or in 50 mM 3-AT pre-treated erythrocytes (^***^, *p* < 0.001, Table 1).

### 3.2. Oxidative Conditions Assessment

#### 3.2.1. TBARS Levels

**Figure 3:**
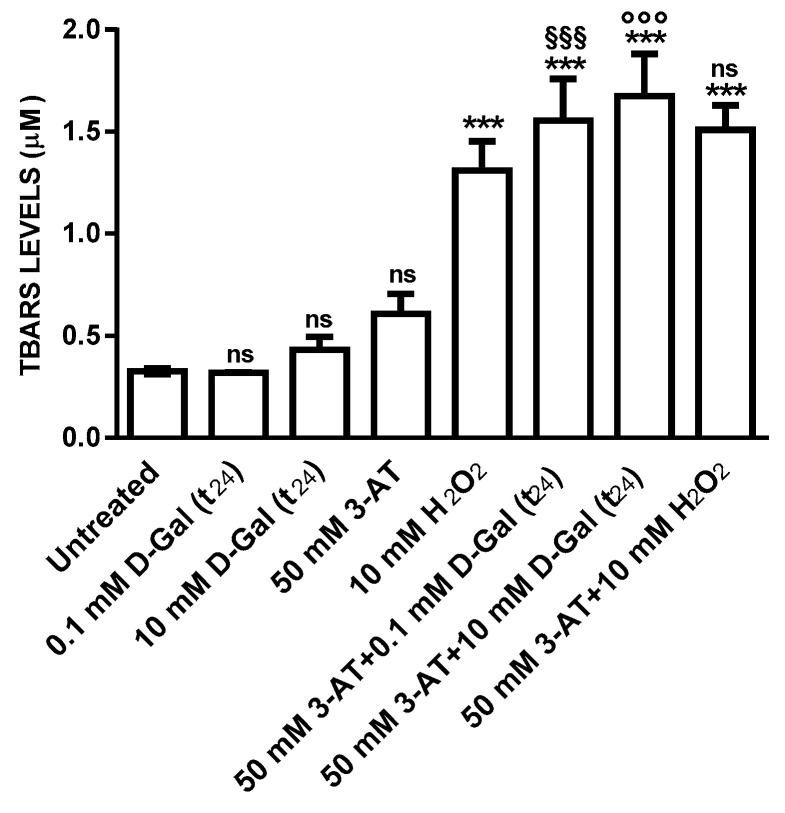
TBARS levels (µM) in untreated (control) and in 0.1 and 10 mM d-Gal (t_24_)-treated erythrocytes, with or without 50 mM 3-AT preincubation. ns, not significant versus control or 10 mM H_2_O_2_; ^***^, *p* < 0.001 versus control, ^§§§^ p < 0.001 versus 0.1 mM d-Gal (t_24_) and ^°°°^, *p* < 0.001 versus 10 mM d-Gal (t_24_) as determined by one way ANOVA followed by Bonferroni’s post hoc test (*n* = 6).

Thiobarbituric-acid-reactive substances (TBARS) levels measured in erythrocytes after a 3 (data not shown) and 24 h incubation with 0.1 or 10 mM d-Gal and in 50 mM 3-AT-incubated erythrocytes were comparable to those detected in control erythrocytes (Figure 3). After 1 h treatment of erythrocytes with 10 mM H_2_O_2_, known to produce lipid peroxidation, TBARS levels were significantly higher than those measured in control and after 3 (data not shown) or 24 h treatment with d-Gal. TBARS levels in erythrocytes treated with 50 mM 3-AT + 0.1 mM d-Gal (t_24_) or with 50 mM 3-AT + 10 mM d-Gal (t_24_) were significantly higher with respect to those measured in control and 0.1 or 10 mM d-Gal-treated erythrocytes. The latter however, were not significantly different with respect to TBARS levels measured in erythrocytes treated with 10 mM H_2_O_2_ alone.

#### 3.2.2. Membrane Sulfhydryl Groups Content Measurement

**Figure 4:**
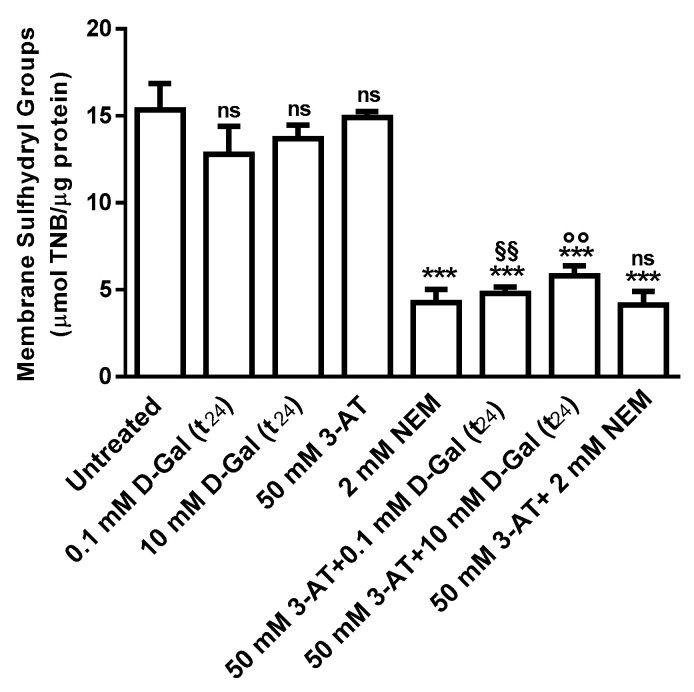
Membrane sulfhydryl groups content (µmol TNB/µg protein) in untreated (control) and in 0.1 or 10 mM d-Gal(t_24_)-treated erythrocytes. ns, not significant versus control or 2 mM NEM, ^***^, *p* < 0.001 versus control, ^§§^, *p* < 0.01 versus 0.1 mM d-Gal (t_24_) and ^°°^, *p* < 0.01 versus 10 mM d-Gal (t_24_) as determined by one way ANOVA followed by Bonferroni’s post hoc test (*n* = 6).

Membrane sulfhydryl groups content (µmol TNB/µg protein) measured after a 3 (data not shown) and 24 h (Figure 4) incubation of erythrocytes with 0.1 or 10 mM d-Gal and in 50 mM 3-AT-incubated erythrocytes was not significantly different with respect to those measured in control. As expected, after 1 h of erythrocytes treatment with the alkylating compound NEM (2 mM), sulfhydryl groups content was significantly lower than control. Levels of membrane sulfhydryl groups in erythrocytes treated with 50 mM 3-AT plus 0.1 or 10 mM d-Gal (t_24_) were significantly reduced with respect to control and 0.1 or 10 mM d-Gal (t_24_) alone. Erythrocytes treated with 50 mM 3-AT plus 2 mM NEM showed sulfhydryl groups levels lower than control, but not significantly different with respect to 2 mM NEM alone after a 3 (data not shown) and 24 h incubation.

#### 3.2.3. Methemoglobin Measurement

**Figure 5:**
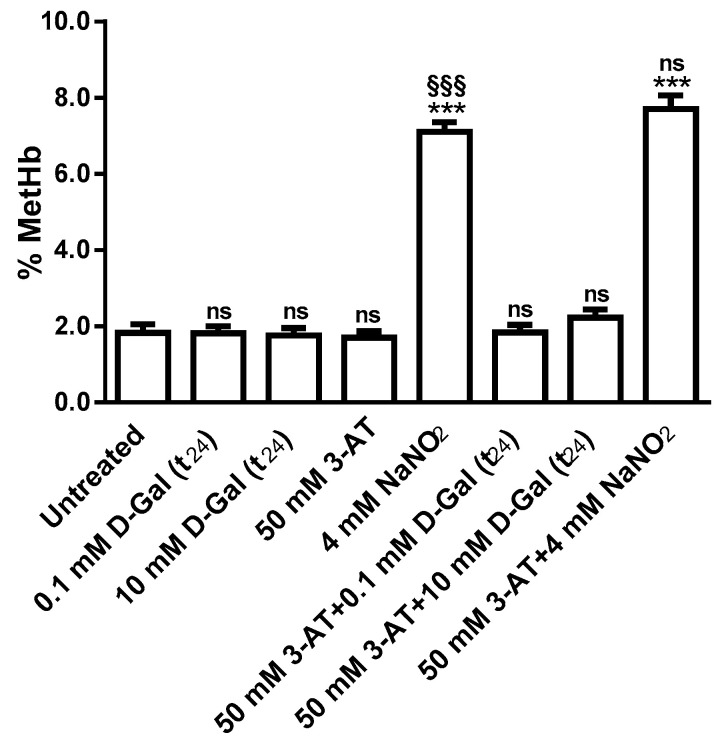
MetHb levels (%) in untreated (control) and in 0.1 or 10 mM d-Gal(t_24_)-treated erythrocytes with or without 50 mM 3-AT. ns, not significant versus untreated erythrocytes (control) and 4 mM NaNO_2_, ^***^, *p* < 0.001 versus control, ^§§§^, *p* < 0.001 versus 0.1 or 10 mM d-Gal (t_24_) with or without 50 mM 3-AT pre-incubation as determined by one way ANOVA followed by Bonferroni’s post hoc test (*n* = 6).

Figure 5 shows Methemoglobin levels (% MetHb) in erythrocytes treated with different d-Gal concentrations (0.1 and 10 mM, 24 h incubation) with or without 50 mM 3-AT pre-treatment. MetHb levels measured after 3 h (data not shown) and after 24 h were not significantly different with respect to those detected in untreated erythrocytes (control). After 1 h of erythrocytes treatment with 4 mM NaNO_2_, a well-known MetHb-forming agent, MetHb levels (%) were significantly higher than control. Erythrocytes treated with 50 mM 3-AT plus 4 mM NaNO_2_ showed MetHb levels (%) higher than control, but not significantly different with respect to 4 mM NaNO_2_ alone after both a 3 (data not shown) and 24 h incubation.

### 3.3. Band 3 Protein Expression Levels Determination

**Figure 6:**
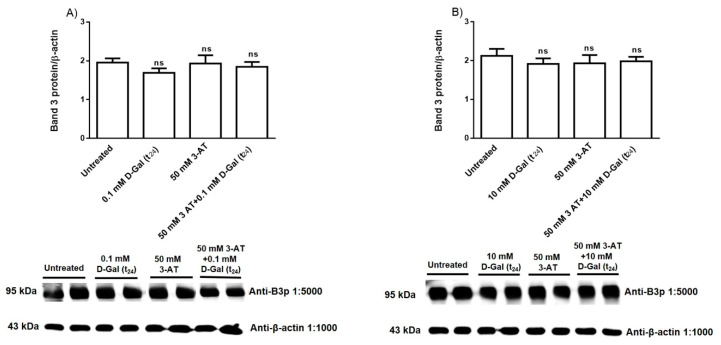
Band 3 protein expression levels measured in untreated (control) and in 0.1 (**A**) or 10 mM (**B**) d-Gal (t_24_)-treated erythrocytes, with or without 50 mM 3-AT pre-incubation, detected by Western blot analysis. ns, not significant versus untreated (control), as determined by one-way ANOVA followed by Bonferroni’s post hoc test (*n* = 5).

Figure 6A,B show B3p expression levels in erythrocytes incubated for 24 h with 0.1 or 10 mM d-Gal respectively, with or without pre-incubation with 50 mM 3-AT. Proteins expression after 3 (data not shown) and 24 h incubation was not significantly different with respect to that determined in untreated erythrocytes (control).

### 3.4. Glycated Hemoglobin Measurement

**Figure 7:**
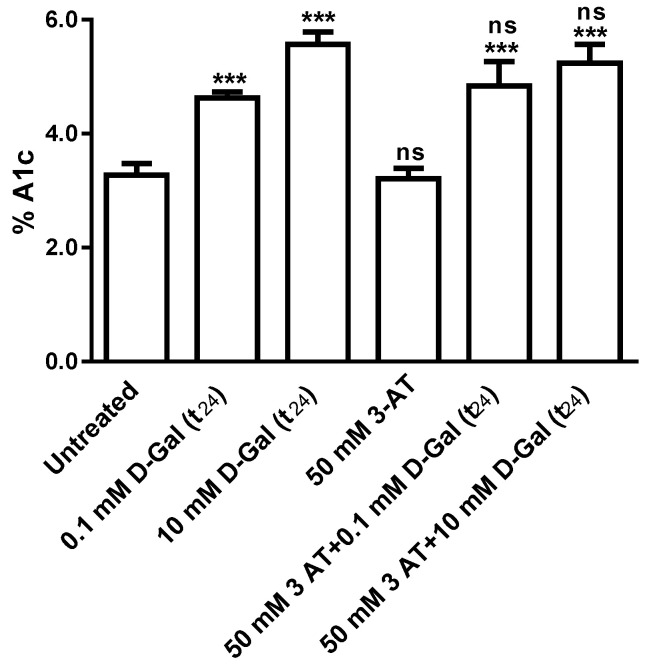
Glycated hemoglobin measurement (% A1c) in d-Gal-treated erythrocytes incubated for 24 h with different d-Gal concentrations (0.1 or 10 mM) with or without 50 mM 3-AT, ^***^, *p* < 0.001 versus untreated (control); ns, not significant versus both 0.1 mM d-Gal or 10 mM d-Gal, as determined by one way ANOVA followed by Bonferroni’s post hoc test (*n* = 10).

Figure 7 shows glycated hemoglobin levels (% A1c) measured in erythrocytes treated with different d-Gal (0.1 and 10 mM) concentrations for 24 h, with or without 50 mM 3-AT treatment. %A1c levels were unchanged after 3 h exposure to d-Gal (data not shown), while, after 24 h, they were significantly increased with respect to control. Treatment with 50 mM 3-AT alone did not change %A1c levels with respect to those measured in untreated erythrocytes, while %A1c levels in erythrocytes treated with 50 mM 3-AT plus d-Gal (0.1 or 10 mM) were not significantly different with respect to those of erythrocytes treated with d-Gal alone and higher than control.

## 4. Discussion

Chronic administration of d-Gal has been widely used as a model to mimic a process very similar to the natural aging, provoking oxidative stress [49] via increased production of ROS and changes in antioxidant enzyme activities in the cell [50]. The decline in cellular homeostatic redox capacity is responsible for macromolecules oxidation, thus compromising their functions [51]. ROS generation may inflict extensive damage to the erythrocyte membranes due to membrane proteins oxidation and lipid peroxidation [52], thus inducing membrane fluidity alteration, which reflects not only on membrane bilayer, but also on intracellular environment. In this regard, specific modified forms of hemoglobin (hemicromes) [53] have been shown to bind to membrane proteins, determining notable alteration of membrane structure, namely on senescent erythrocytes [19].

Though several in vivo experiments have been conducted on plasma or blood cells from animal models [6,18,19,20,21], the action mechanism of excessive d-Gal on human erythrocytes remains poorly clarified. d-Galactose-induced superoxide production implies H_2_O_2_ formation due to superoxide dismutase (SOD). As a consequence, H_2_O_2_, if abnormally accumulated, can inflict damage to the cell. Under normal conditions, the entire endogenous antioxidant system of erythrocytes, involving catalase, SOD and glutathione peroxidase (GPx) contributes to neutralize H_2_O_2_, thus minimizing its detrimental oxidative effects. Nevertheless, an unbalance between antioxidant enzymes involved in cell defense against ROS overproduction seems to be a mechanism through which d-Gal induces cell senescence [19]. The re-organization of cell proteins under d-Gal treatment has been also considered as an effect of cell aging. In particular, the oxidant effects of aging on Hb and membrane transport systems have been studied [54,55].

As previously stated, d-Gal oxidant effects have been mainly studied in vivo, while, with regard to in vitro investigations, there is still a lack of knowledge. A few data are available from an in vitro study by Delwing-de Lima and coauthors using d-Gal at final concentrations of 0.1, 0.3, 0.5 and 10 mM on blood withdrawn from 30- or 60-day-old rats. These authors consider d-Gal concentrations comprised in a range including both physiological and pathological values. According to other authors, the normal range in human healthy adults corresponds to 0.000012 mM, which is lower than what measured in newborn plasma [3,4]. On this basis, the aim of the present investigation was to verify the effect of d-Gal at high concentrations (0.1 or 10 mM) in in vitro human erythrocytes model. In particular, the purpose was to evaluate anion exchange capability through B3p and to verify the potential mechanism through which d-Gal may affect this function. As reported elsewhere, B3p is essential to erythrocytes homeostasis, whose assessment is a valid tool to detect damage in case of oxidative stress-related diseases, such as Systemic Sclerodermia [56], Canine Leishmaniasis [45] and hyperglycemic conditions [33], or in other oxidative conditions modeled in vitro [27,34].

The first step of the present research was to evaluate the SO_4_^2−^ uptake through B3p [27,31,32] after either 3 or 24 h treatment with d-Gal (0.1 and 10 mM). Under these experimental conditions, the rate constant for SO_4_^2−^ uptake. It is reasonably to suggest that, as a consequence of a reduced rate for SO_4_^2−^, the amount of this anion trapped after 45 min of incubation in SO_4_^2−^ medium was, in turn, significantly reduced (Figure 2A,B). Similar d-Gal concentrations have been applied by Delwing-de Lima and coauthors, which observed a consequent protective increase in catalase activity in both young and old rat’s erythrocytes. In this regard, to verify whether the d-Gal effect was mitigated by the endogenous antioxidant system, human erythrocytes were preventively exposed to 50 mM 3-AT, a specific catalase inhibitor [37]. In these conditions, the d-Gal (0.1 and 10 mM)-induced reduction in the rate constant for SO_4_^2−^ uptake was exacerbated, suggesting that antioxidant enzymes, namely catalase, are critically involved in limiting the effect of excessive d-Gal.

The above mentioned results indicate that the modification of anion exchange capability through B3p in erythrocytes is associated to an increase of oxidative stress. Hence, to better explore this effect and according to what reported by other authors [19,57], lipid peroxidation and oxidation of membrane sulfhydryl groups mainly belonging to B3p [58] have been successively evaluated. This choice is also related to our previous studies on the impact of oxidant conditions on B3p efficiency [27,34,45,59,60]. As shown by the present results, d-Gal effect was mediated neither by membrane lipids nor by membrane sulfhydryl groups oxidation, at any concentration and at any time of incubation. Also in this case, the use of the catalase inhibitor before d-Gal incubation produced a significant oxidation of both lipids and membrane proteins (Figure 3 and Figure 5). As a further step, to better clarify the mechanism of action of d-Gal in human erythrocytes, B3p protein abundance has been also determined (Figure 5 and Figure 6), showing that in d-Gal (0.1 or 10 mM)-treated erythrocytes, at both t_3_ and t_24_, the expression levels of B3p were not reduced.

Therefore, following brief exposure of erythrocytes to high concentrations of d-Gal, the anion exchange capability through B3p is lowered, independently on TBARS production, or alteration in oxidative state, or expression levels of the membrane protein responsible for anion exchange. These results are similar to what previously reported by our group on human erythrocytes exposed in vitro to H_2_O_2_ [27]. Recent scientific evidences have reported that d-Gal accumulation induces oxidative stress, cell senescence and cytotoxicity [61]. In this regard, B3p functions, being crucial in the systemic homeostasis maintenance and affected by oxidative stress, could be correlated to oxidative stress-induced alterations during galactosemia. The hypothesis was that the crosslink between cytoplasmatic domain of B3p and Hb [62] was somehow compromised, thus reflecting on the efficiency of anion exchange, which was lowered. Such change seems not to impair the binding of anti B3p antibody.

This prompted us to take into account Hb in the present investigation, with specific regard to oxidation and glycation processes. In this respect, our results demonstrated that 3 h of incubation with d-Gal (0.1 or 10 mM) are insufficient to induce oxidative damage or glycation processes on Hb (data not shown). On the other hand, a longer incubation (24 h) with d-Gal does not induce oxidative stress events (Figure 5), but allows for the formation of higher glycated Hb levels (%A1c), with respect to untreated erythrocytes (Figure 7). Hence, we could suggest that, unlike what already shown in erythrocytes exposed in vitro to high glucose concentrations [33], d-Gal could generate early Hb glycation [63], which induces direct damage on Hb and, in turn, affects the exchange capability via B3p, cross-linked to Hb. However, we recently reported that in vitro exposure of human erythrocytes to high glucose induces an acceleration of the rate constant of SO_4_^2−^ uptake, without A1c formation [33], contrarily to what observed here following d-Gal treatment. This discrepancy could be due to the different action mechanism of the two monosaccharides. Nevertheless, determination of anion exchange capability, in both cases, can be considered a very sensitive tool to detect the effects of exposure to high sugars concentrations [33], which were not revealed by parameters currently used to verify oxidative damage at either lipid or protein level, i.e., TBARS formation, oxidation and expression levels of membrane proteins.

According to other investigations, it seems more likely that in the present experimental conditions glycation of Hb rather than of B3p may occur, putatively due to a faster penetration of d-Gal through erythrocytes membrane at pH 7 than glucose [64] (approximately 3 times), which would let d-Gal induce glycation of Hb more rapidly than glycation of membrane proteins such as B3p. In addition, glycation of B3p should be excluded, since expression levels of this protein are unchanged after d-Gal treatment and, as reported elsewhere [60], the migration of glycated forms of proteins is usually slower. Kenawy and collaborators [63], using d-Gal treatment as a model of aging, stated that 90 days of d-Gal treatment are needed to observe increased levels of glycated Hb in vivo. Therefore, the present study provides novel elements about the first impact of excessive d-Gal on erythrocytes, which, as cited above, seems not associated to oxidative damage but rather to glycation events. In this regard, other authors have investigated proteins glycation induced by different sugars. In particular, the evidence of a d-Gal-dependent glycation has been already provided by Ledesma-Osuna and collaborators [65], reporting that d-Gal is more reactive than glucose or d-lactose, leading to the coupling of 10, 3 and 1 sugar residues, respectively, after 120 min of reaction on bovine serum albumin. Consequently, with regard to the present data and to the present experimental conditions, it is reasonable to suggest that the high reactivity of d-Gal could promote glycation as an early effect.

What recently reported by our group [32] describes that erythrocytes exposed to high glucose or erythrocytes withdrawn from patients with poorly controlled hyperglycemia exhibited an accelerated rate constant of SO_4_^2−^ uptake, with the difference that only in the latter condition A1c formation was seen. In light of this report, and taking into account that oxidative conditions along with glycation affect erythrocytes in diabetic patients, it is reasonable to suggest that the extent of A1c formation observed here after d-Gal treatment is not sufficient to provoke an increase in anion exchange capability through B3p comparable to those detected in diabetic patients, but represents a first step of glycation process, which in any case alters the efficiency of the anion exchanger, lowering its rate.

It is interesting to point out that, according to Kenawy [63] and co-authors, 90 days of d-Gal treatment finally led to insulin resistance, which explains oxidative stress and inflammation at brain level. These authors further underscored that insulin resistance reduced glucose utilization, leading to mitochondrial dysfunction and ROS production. As in the present investigation d-Gal exposure was shorter, we could hypothesize that glycation occurred as the first process in our conditions, followed by oxidative stress, both leading to a detrimental effect on B3p. In addition, GSH/GSSG ratio considered as an indicator of oxidative stress is not altered by d-Gal treatment (Figure S1).

At any rate, the in vitro oxidant effect of d-Gal cannot be excluded and higher d-Gal concentrations and longer time of incubation are probably needed to induce oxidative stress events in human erythrocytes, as demonstrated by other studies on senescence models [19,66]. As reported elsewhere, the activities of some anti-oxidants enzymes, such as SOD1 and catalase, decline during aging [67] and, according to other authors, this decrease may depend on an increase in glycation of SOD [68], with consequent increased production of free radicals, responsible for aging. Such observation may further support the hypothesis that, under excessive d-Gal concentrations, oxidative stress follows glycation events. In this latter case, Jafarnejad and collaborators have demonstrated that a reduction in protein glycation can be prevented by acetyl salicylic acid (ASA) [69], known as inhibitor of glycation, thus inducing a decrease in AGEs levels and A1c formation [70]. Therefore, in analogy to what reported by these authors, ASA could be also used to prevent the elevation of glycation levels, specifically for Hb glycation in case of high d-Gal concentrations.

The contribution of the antioxidant system in preventing oxidative damage after production of glycated proteins during d-Gal treatment of erythrocytes is not fully understood [19,71] and future studies will be needed to elucidate the link between glycation and oxidative events.

Finally, it is opportune to mention some limits of this investigation, as well as future perspectives. As a whole, the present study reports for the first time the effect of high d-Gal on erythrocytes by using a validated in vitro model monitoring the anion exchange capability through B3p. At a first stage, high d-Gal seems to reduce the anion exchange capability of erythrocytes as a consequence of Hb glycation, rather than by oxidative action, which can only be detected after inhibition of catalase. This important finding underscores that d-Gal effectively induced oxidative stress, which, however, was mitigated by catalase activity. This would suggest that in vivo and, more specifically, in those pathological conditions characterized by absence or reduction of functioning catalase, the effects of the oxidative action of d-Gal could be more relevant. Future studies should focus on the impact of d-Gal in subjects having a deficiency in erythrocytes catalase such as, for example, patients suffering of acute myocardial infarction. In this context, the use of antioxidants chosen among the most lipophilic radical-scavenging antioxidants, i.e., alpha-tocopherol [72], or low molecular weight antioxidants, such as vitamin C and polyphenols, should also be investigated.

In addition, techniques more sensitive than those employed here (i.e., HPLC to detect MDA levels) to assess the oxidative impact of d-Gal should be also considered, in an attempt to give more details about high d-Gal toxicity and having the present outcomes as a starting point useful to shed light on this topic.

## 5. Conclusions

Taken together, our findings suggest that: i) B3p function assessment is a sensitive tool to verify the impact of d-Gal on erythrocytes homeostasis; ii) d-Gal (0.1 and 10 mM) induces a reduction in anion exchange capability independent from membrane lipids peroxidation, membrane sulfhydryl groups oxidation, MetHb formation, or changes in B3p expression levels; iii) d-Gal effect, reflecting on B3p efficiency, could develop through a two-phases process, with the first associated to early glycation of Hb, overwhelming the oxidant effect of d-Gal; iv) d-Gal-induced oxidative damage could not be excluded, but is promptly mitigated by the endogenous antioxidant system, specifically catalase.

Finally, our investigation adds first elements to the knowledge about d-Gal toxicity namely at membrane transport systems levels, which is not only associated to in vitro aging models, but also to failure of d-Gal metabolism in humans, due to congenital deficiency of enzymes, leading to d-Gal accumulation. This study could offer a good basis to understand the early effect of excessive d-Gal, shedding more light on possible complications related to undiagnosed galactosemia and highlighting the relationship between AGEs and oxidative events, which need to be better clarified, as possibly involved in the onset of galactosemia symptoms

## Figures and Tables

**Table 1:** Rate constant for SO_4_^2−^ uptake and amount of SO_4_^2−^ trapped in untreated and in d-Gal (t_3_ or t_24_)-treated erythrocytes at different concentrations (0.1 or 10 mM), with or without 3-AT pre-treatment.

	Rate Constant (min^−1^)	Time (min)	*n*	SO_4_^2−^ Amount Trapped after a 45 min Incubation in SO_4_^2−^ Medium [SO_4_^2−^] L cells x10^−2^
untreated (control)	0.067 ± 0.001	14.92	8	313.81 ± 11.09
0.1 mM d-Gal (t_3_)	0.066 ± 0.001 ^**^	15.15	6	269.76 ± 39.74 ^**^
0.1 mM d-Gal (t_24_)	0.065 ± 0.001 ^***^	15.38	6	203.16 ± 39.74 ^***^
10 mM d-Gal (t_3_)	0.060 ± 0.001 ^***^	16.66	6	191.26 ± 19.90 ^***^
10 mM d-Gal (t_24_) 50 mM 3-AT	0.051 ± 0.001 ^***,ns^ 0.068 ± 0.001 ^ns^	19.60 14.70	6 6	179.97 ± 7.23 ^***^ 343.43 ± 12.23 ^ns^
50 mM 3-AT + 0.1 mM d-Gal (t_3_)	0.065 ± 0.001 ^***,§§§^	15.38	6	146.85 ± 18.46 ^§§§^
50 mM 3-AT + 0.1 mM d-Gal (t_24_)	0.066 ± 0.001 ^***,°°°^	15.15	6	191.26 ± 15.21 ^°°°^
50 mM 3-AT + 10 mM d-Gal (t_3_)	0.040 ± 0.001 ^***^, ^ns^	25.00	6	172.85 ± 18 ^ns^
50 mM 3-AT + 10 mM d-Gal (t_24_)	0.038 ± 0.001 ^***,§^	26.31	6	152.85 ± 15.87 ^§^
10 µM DIDS	0.018 ± 0.001 ^***^	55.5	10	4.75 ± 8.50 ^***^

Time is the reciprocal of the rate constant (see Materials and Methods for definition). Data are presented as means ± SEM from separate *n* experiments, where *ns* is not significant versus untreated*,*10 mM d-Gal (t_3_) and 50 mM 3-AT + 10 mM d-Gal (t_24_) or 10 mM d-Gal (t_3_) and 50 mM 3-AT + 10 mM d-Gal (t_24_);^**^, p < 0.01 versus untreated (control); ^***^, p < 0.001 versus control, *^§^, p* < 0.05 significant versus 50 mM 3-AT + 10 mM d-Gal (t_3_); *^§§§^, p* < 0.001 versus 0.1 mM d-Gal (t_3_) and, *^°°°^, p* < 0.001 versus 0.1 mM d-Gal (t_24_), as determined by one way ANOVA followed by Bonferroni’s post hoc test (*n* = 6).

## Supplementary Materials

The following are available online at https://www.mdpi.com/2076-3921/9/8/689/s1, Figure S1: Estimation of GSH/GSSG ratio measured in untreated (control) and d-Gal treated erythrocytes at different concentrations (0.1 and 10 mM) with or without 50 mM 3-AT incubated for 24 h. *ns* not significant *vs* control; **** p < 0.001* significant *vs* control; *^§§§^ p < 0.001 vs* 0.1 mM d-Gal; *^°°°^ p < 0.001* significant *vs* 10 mM d-Gal, as determined by one way ANOVA followed by Bonferroni’s *post hoc* test (*n = 3*).
