# Supplementary figures and images for: d-Galactose Decreases Anion Exchange Capability through Band 3 Protein in Human Erythrocytes
Source: Antioxidants (Basel). 2020 Aug 2;9(8):689. doi: 10.3390/antiox9080689 (PMC7465100; doi:10.3390/antiox9080689)

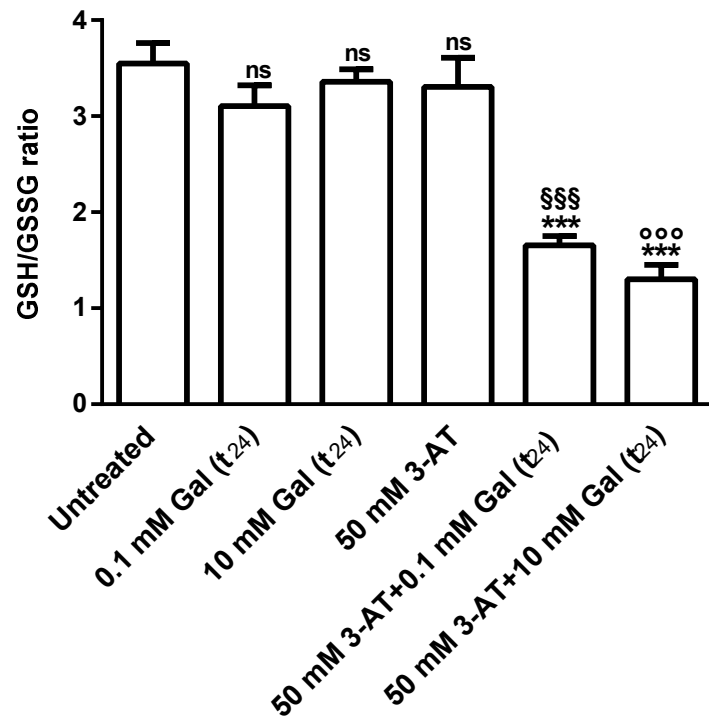

Figure S1.

Supplement: Supplementary file 1 [file antioxidants-09-00689-s001.pdf]
